# Clinical Outcomes of Pterygoid and Maxillary Tuberosity Implants: A Systematic Review

**DOI:** 10.3390/jcm13154544

**Published:** 2024-08-03

**Authors:** Kami Raouf, Bruno Ramos Chrcanovic

**Affiliations:** 1 Faculty of Odontology, Malmö University, 214 21 Malmö, Sweden; kamiarkami@gmail.com; 2 Department of Oral and Maxillofacial Surgery and Oral Medicine, Faculty of Odontology, Malmö University, 214 21 Malmö, Sweden

**Keywords:** dental implant, pterygoid process, maxillary tuberosity, failure, survival analysis, systematic review

## Abstract

**Background:** This study aimed to assess the survival of implants placed in the maxillary tuberosity or in the pterygomaxillary region of the maxilla, based on a systematic review of the literature. **Methods:** An electronic search was undertaken in three databases. The cumulative survival rate (CSR) was calculated. The log-rank (Mantel–Cox) test was used to compare the survival distributions between some groups. **Results:** Thirty-eight studies were included, reporting 3446 implants (3053 pterygoid, 393 tuberosity) in 2245 patients, followed up for a mean ± SD of 61.0 ± 36.3 months (min–max, 1–144). A total of 208 pterygoid and 12 tuber implants failed, with a clear concentration of failures in the first year of follow-up and a 10-year CSR of 92.5% and 96.9%, respectively. The survival of pterygoid implants was lower than that of implants in the maxillary tuberosity (*p* = 0.006; log-rank test), and the survival of implants submitted to early/delayed loading was lower than that of immediately loaded implants (*p* < 0.001; log-rank test). Non-splinted implants presented higher failure rates. Few cases of intra- or postoperative complications were reported. **Conclusions:** Implants placed in the pterygoid process/maxillary tuberosity present a high 10-year CSR, although with lower survival for pterygoid in comparison to tuber implants. Pterygoid/tuber implants that are splinted with other implants may present higher survival rates than those that are not splinted.

## 1. Introduction

The placement of dental implants in the posterior maxillary area has been considered problematic, as this is a region where the bone is typically characterized by low-density trabecular bone and thin cortical bone. These features may increase the risk of implant failure [1]. Moreover, the presence of the maxillary sinus often compromises the volume of bone available in the region [2]. This is aggravated by a process called pneumatization, which is the progressive expansion of sinus volume, a process that begins after birth and carries on until the second and third decades of life in women and men, respectively [3]. The extraction of posterior maxillary teeth makes the matter worse, as not only does additional pneumatization occur [4], but the alveolar bone is resorbed and loses height [5].

Maxillary sinus elevation surgery, also called maxillary sinus lift or augmentation, is an option of treatment for when rehabilitation of the edentulous posterior maxilla with implants is desirable. In this technique, graft material is added to the floor of the sinus to increase the height of available bone for implant placement [6]. Complications can, however, occur, and can include the tearing of the (Schneiderian) sinus membrane, bleeding, infection, loss of graft material, and persistent sinusitis, among others [7].

Another option consists of an interpositional bone graft after a Le Fort I osteotomy. The surgical technique may help to correct the vertical and horizontal jaw relations in patients with edentulous and atrophic jaws [8]. The approach is, however, very invasive and traumatic, and possible complications include wound-healing disturbance, transient oroantral fistula, and partial graft losses due to infection and sequestration [9].

A titanium mesh can be customized with computer-aided design–computer-aided machining (CAD-CAM). It can be fixated in the maxilla and loaded with graft material in order to increase maxillary bone volume. The technique has the advantage of augmenting the atrophic maxilla according to a pre-established shape. Yet, the approach presents a high prevalence of mesh exposure due to the flap tension that is usually applied to close the surgical field, which may result in mucosal rupture and potentially in infection [10].

The placement of short implants is an alternative when not so much bone is available. However, there is a recommendation concerning the minimum amount of bone in which an implant should be anchored to increase the possibility of achieving primary stability and minimize the risk of early implant loss, which is 4 mm [11], and this is not always the case. Moreover, short implants have a higher risk of failure in comparison to longer implants [12].

Other option is the placement of zygomatic implants, which are anchored directly in the zygomatic bone, avoiding grafting procedures [13]. The procedure is very invasive and traumatic, though, with the potential postoperative occurrence of sinusitis, soft tissue infection, and oroantral fistulas, and even the use of surgical drill guides to help in the accuracy of the implant position presents the risk of serious injury to important anatomical structures [14].

Then, there is the alternative of placing implants in the maxillary tuberosity or in the pterygomaxillary region, which provide bone anchorage in the posterior maxilla without the need for sinus augmentation or supplemental grafting [15], providing support and retention for implant restoration and eliminating posterior cantilevers [16]. Engaging the cortical bone of the pterygoid plate with long implants can improve primary stability, thereby facilitating long-term success [15]. But as with any other surgical procedure, the approach is also subjected to complications, such as the displacement of the implant into the pterygoid fossa [17], intraoperative venous bleeding, and trismus [18]. And the technique may be contraindicated in cases of absence of enough bone in the tuberosity area, the presence of impacted third molars, and in patients with reduced mouth opening [19].

The purpose of the present study was to assess the survival of implants placed in the maxillary tuberosity or in pterygomaxillary region of the maxilla based on a systematic review of the literature.

## 2. Materials and Methods

This study followed the PRISMA 2020 Statement guidelines [20]. Registration in PROSPERO was undertaken with the registration number CRD42023492041.

### 2.1. Focused Question

The focused question was elaborated by using the PICO format (participants, interventions, comparisons, outcomes): What is the survival rate of pterygoid or maxillary tuberosity implants in patients being rehabilitated with implant-supported prostheses?

P: Patients rehabilitated with prostheses supported by pterygoid or maxillary tuberosity implants.

I: Rehabilitation with pterygoid or maxillary tuberosity implants.

C: Comparison between pterygoid and maxillary tuberosity implants.

O: Implant survival.

### 2.2. Search Strategies

An electronic search without time restrictions was firstly undertaken in October 2022, last updated with a complementary search in October 2023, in the following databases: PubMed/Medline, Web of Science, and Science Direct. The following terms were used in the search strategies:

(dental implant OR oral implant OR pterygoid implant) AND (pterygoid OR maxillary tuber OR maxillary tuberosity OR pterygomaxillary).

A manual search of dental implants-related journals, including Clinical Implant Dentistry and Related Research, Clinical Oral Implants Research, European Journal of Oral Implantology, Implant Dentistry, International Journal of Implant Dentistry, International Journal of Oral and Maxillofacial Implants, International Journal of Oral Implantology, International Journal of Prosthodontics, Journal of Clinical Periodontology, Journal of Oral Implantology, Journal of Periodontology, Journal of Prosthetic Dentistry, Journal of Prosthodontics, and Journal of Prosthodontic Research was performed. The reference list of the identified studies and the relevant reviews on the subject were also checked for possible additional studies.

The grey literature was also searched, including ClinicalTrials.gov, the International Clinical Trials Registry Platform (https://trialsearch.who.int/), Open Grey (https://opengrey.eu/), and the EU Clinical Trials Register (https://www.clinicaltrialsregister.eu/).

### 2.3. Inclusion and Exclusion Criteria

The eligibility criteria included clinical human studies, either randomized or not, reporting cases of patients that received implants in the maxillary tuberosity or in the pterygoid process. Case reports were also considered, provided that follow-up information was reported. Only cases rehabilitated with cylindrical screw-type modern dental implants of titanium (commercially pure titanium; c.p.Ti) or its alloys were considered. The exclusion criteria were technical reports, animal studies, in vitro studies, and review papers.

### 2.4. Study Selection

The titles and abstracts of all reports identified through the electronic searches were read independently by two reviewers (K.R., B.R.C.). For studies appearing to meet the inclusion criteria, or for which there were insufficient data in the title and abstract to make a clear decision, the full report was obtained. Disagreements were resolved by discussion between the authors.

RefWorks Reference Management Software (Ex Libris, Jerusalem, Israel) was used in order to detect duplicate references in different electronic databases.

### 2.5. Quality Assessment

The quality assessment of the included case report publications was carried out according to the 13-item CARE guidelines [21]. A score of 1 was given for each item outlined in the CARE guidelines, with a maximum score of 30 per case report. A score of 30 represents the highest quality, and a score of two-thirds or more of the points is considered high quality.

The quality assessment of the included case series was carried out according to the Quality Assessment Tool of the National Institutes of Health [22]. The NIH quality assessment tool calculates study quality on the basis of nine criteria. The ratings on the different items were used by the reviewers to assess the risk of bias in the study due to flaws in study design or implementation. The studies were classified as “good”, “fair”, or “poor” quality. In general terms, a “good” study has the least risk of bias, and its results are considered to be valid. A “fair” study is susceptible to some bias, which is deemed not sufficient to invalidate its results. The fair quality category is likely to be broad, so studies with this rating will vary in their strengths and weaknesses. A “poor” rating indicates a significant risk of bias. Studies of “good” quality were judged to have at least 7 points.

The reviewers went together through all the items of these quality assessment tools, as an initial calibration, after which the quality assessment was carried out independently by the reviewers. Any disagreement was resolved by discussion between the authors.

### 2.6. Definitions

A pterygoid implant was defined as an implant engaging in the cortical bone formed by the posterior wall of the maxillary tuberosity, the horizontal process of the palatine bone, and the pterygoid process of the sphenoid bone [23].

An implant placed in the maxillary tuberosity was defined as one placed in the maxillary tuberosity without directly engaging the high superior portion of the pterygoid plate of the sphenoid bone [24].

An implant was considered a failure if the presenting signs and symptoms led to implant removal, i.e., a lost implant. Implant failure could be either early (due to the inadequacy of the host to establish or promote osseointegration in the early stages of healing) or late (due to the failure of either the established osseointegration or function of dental implants) [25]. The fracture of an implant was also considered a failure.

### 2.7. Data Extraction

From the studies included in the final analysis, the following data were extracted (when available): year of publication, study design, country, study setting, number of patients, patients’ age and sex, implant healing period, implant location (pterygoid process or maxillary tuberosity), total number of placed implants, number of failed implants, type of prosthetic rehabilitation, presence of smokers in the patients’ study group, and follow-up time. Authors were contacted and asked to provide missing data.

### 2.8. Analyses

The mean, standard deviation (SD), and percentage were calculated for several variables. The tests performed were the following: Kolmogorov–Smirnov (to evaluate the normality of the distribution), Levene’s test (to evaluate homoscedasticity), Student’s *t*-test or Mann–Whitney (for two independent groups, continuous variables), and Pearson’s chi-squared or Fisher’s exact test (for categorical variables). The interval survival rate (ISR) of implants was calculated using the information for the period of failure extracted from the included studies, and the cumulative survival rate (CSR) was calculated over the maximal period of follow-up reported, in a life table survival analysis. The log-rank (Mantel–Cox) test was used to compare the survival distributions between pterygoid and tuber implants. The degree of statistical significance was considered *p* < 0.05. All data were statistically analyzed using the Statistical Package for the Social Sciences (SPSS) version 28 software (SPSS Inc., Chicago, IL, USA).

## 3. Results

### 3.1. Literature Search

**Figure 1:**
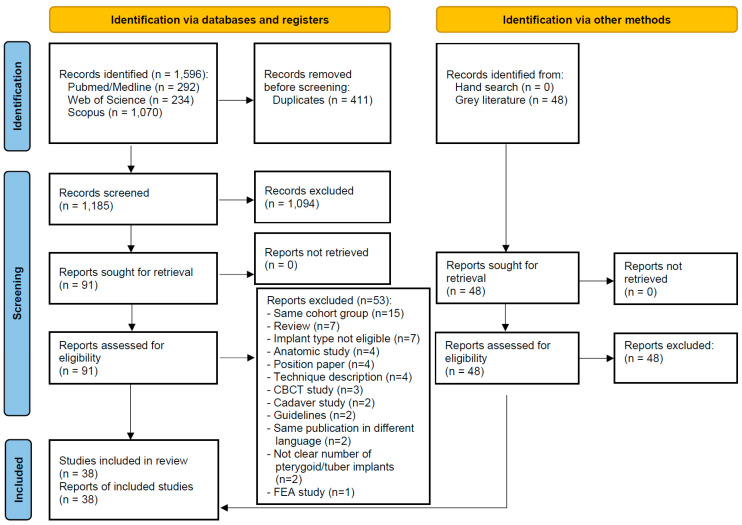
Study screening process (CBCT—Cone Beam Computed Tomography, FEA—Finite Element Analysis).

The study selection process is summarized in Figure 1. The search strategy in the databases initially resulted in 1596 papers (292 in PubMed/Medline, 234 in Web of Science, 1070 in Scopus), and 48 entries were found in the searched sources of grey literature. In the end, 38 studies were included in this review.

### 3.2. Description of the Studies

Detailed data on every publication are presented in Table S1, in the Supplementary Materials. Table 1 presents the summarized global data of the included studies, also separately between pterygoid and tuberosity implants.

The 38 included publications [15,17,26,27,28,29,30,31,32,33,34,35,36,37,38,39,40,41,42,43,44,45,46,47,48,49,50,51,52,53,54,55,56,57,58,59,60,61] reported 3446 pterygoid/tuber implants in 2245 patients, of whom 879 were men and 1148 were women, with no information on the sex of 218 patients. Most of the reported implants were placed in the pterygoid process (n = 3053; 88.6%), with much fewer placed in the maxillary tuberosity (n = 393; 11.4%). The great majority of the pterygoid implants were used to support a fixed full-arch prosthesis (75.0%), while implants in the tuber were used more often to support a fixed partial prosthesis of 2–6 units (36.6%), followed by the use to support a fixed full-arch prosthesis (32.8%). About one-third of the implants were submitted to immediate loading.

Most of the cases of the different prosthetic rehabilitation types were submitted to non-immediate loading protocols, with the exception of fixed full-arch prostheses (Table 2).

Few cases of intra- or postoperative complications were reported; these included implant displaced into the pterygoid fossa [17], penetration of the maxillary sinus [42], sinus membrane perforation, sinusitis, severe postoperative pain [45], hemorrhage [45,52], transient (4 weeks) hypoesthesia of the palatine nerve [52], pterygomaxillary pain, for which the implant was removed [52], and local suppuration around a lost implant [39], besides some cases of peri-implant mucositis [40,60]. Two cases of acute bilateral maxillary sinusitis were reported in one study [59], although these patients also received zygomatic implants.

### 3.3. Analyses

A total of 208 pterygoid implants failed, with a clear concentration of failures during the first year of follow-up (Table 3). The 10-year CSR was 92.5%. Twelve tuber implants failed, also with a concentration of failures within the first year after implant installation, with a 12-year CSR of 96.9% (Table 4). The Kaplan–Meier curve for the cumulative survival of all implants (pterygoid and tuber) is shown in Figure S1 (see Supplementary Materials). The survival of pterygoid implants was lower than that of implants in the maxillary tuberosity (*p* = 0.006; log-rank test; for Kaplan–Meier curves see Figure S2 in the Supplementary Materials). The survival of implants submitted to early/delayed loading was lower than that of immediately loaded (implants *p* < 0.001; log-rank test; for Kaplan–Meier curves see Figure S3 in the Supplementary Materials).

Survival analyses of the different prosthetic rehabilitations resulted in a 1-year CSR of 76.8% for single crowns, a 12-year CSR of 97.2% for fixed partial prostheses, a 12-year CSR of 92.2% for fixed full-arch prostheses, and a 3-year CSR of 88.6% for overdentures (Tables S2 to S5, respectively—see Supplementary Materials).

### 3.4. Quality Assessment

All included studies were classified as “good” according to the quality assessment tool (see Tables S6 and S7 in the Supplementary Materials). In most cases the main issues in the publications were related to poorly described statistical methods and to the inclusion of non-consecutive patients in the studies.

## 4. Discussion

The purpose of the present review was to assess the survival of implants placed in the maxillary tuberosity or in the pterygomaxillary region of the maxilla. The results showed that pterygoid and tuber implants present high CSR over 10 years, namely, 92.5% and 96.9%, respectively.

The majority of implant failures, irrespective of whether situated in the pterygoid process or tuber, occurred during the initial year following implant installation. Additionally, a high rate of primary failure (up to prosthetic loading) was observed among implants that were not subjected to immediate loading. This finding also gives rise to the question of what factors may have led to the decision not to apply immediate loading. A considerable percentage of implants fail in the early period after implant installation, regardless of the follow-up time [62], and although it may be difficult to point out one or more specific agents, especially in material compiled from the numerous studies included in this review, which present innumerable confounding elements, many factors can have a negative influence on implant survival [63]. Early implant failure could be associated, for example, with smoking habits, the intake of medications, history of periodontal problems, poor bone quality [64], the number of implants, and the location of the implants [65]. Individuals sometimes present several risk factors, and groups of patients are typically heterogeneous with respect to risk factors and susceptibilities [66]. Thus, the specific effect of an individual risk factor could be isolated neither for individual studies nor for the present review. Unfortunately, this makes impossible to properly evaluate the possible effect of each and every factor to the early failure of implants.

Pterygoid implants presented a higher failure rate than tuberosity implants. This may be connected to the local anatomical differences in these implant sites. Implants placed in the maxillary tuberosity do not anchor the pyramidal process of the palatine or the pterygoid plates of the sphenoid bone [48,51], which makes it possible for implants placed in the pterygoid process to be anchored bicortically [15,34]. However, this is where the issue lies, as placing implants in the complex anatomic region of the pterygoid process may be challenging for the surgeon, not only due to difficult surgical access but also to poor bone quality [67,68,69]. Therefore, even though implants placed in the pterygoid process are usually longer than the ones placed in the tuber, the peculiarities of these anatomical sites may exert a higher influence on implant survival than implant length [12].

The results of the survival analyses suggest that prosthetic restorations supported by tuber/pterygoid implants that are not splinted to other implants, namely single crowns and overdentures, present lower cumulative survival rates than the ones for fixed partial or full-arch prostheses. Although the total number of tuber implants used to support single crowns was low, there was a high rate of implant failure for when this type of prosthetic protocol was applied, which might suggest that splinting these pterygoid/tuber implants with more anterior implants by the prosthetic restoration—and therefore installing a partial fixed prosthesis instead of a single crown—may be advantageous. There are biomechanical advantages to splinting implants; it could protect implants from micromotion [70] while also allowing for a more even distribution of occlusal forces [71]. This could also be connected to the observed lower survival of implants when submitted to early/delayed load than to immediate load, as almost the totality of immediately loaded pterygoid/tuber implants were splinted in full-arch fixed prosthesis.

The survival of implants submitted to early/delayed loading was lower than that of implants immediately loaded. This goes against the results of a meta-analysis on the subject, which indicated that failures are 1.78 times more likely to happen when implants are immediately loaded than when implants are not immediately submitted to masticatory load [72]. This could, in theory, be related to a possible higher prevalence of non-splinted types of prostheses (which presented higher implant failure rates in the present study, as aforementioned) submitted to immediate loading. But this was simply not the case. To make matters worse, the number of non-splinted implants was very small. Thus, other factors could have influenced these results. They could be connected to the characteristics of the different implants used in the clinical studies, such as the geometry of the implant body specially designed for critical bone conditions and implant surfaces combined with high insertion torques during bone healing, which may have minimized the risk of early failure of immediately loaded implants [72]. They could also be associated with differences in the technique, skills, and/or judgment of the many surgeons involved in all these different clinical studies, which may have negatively influenced the implant survival rates [73].

The terminology used for the actual position of the implant may cause some confusion. According to Reiser [74], depending on the angle of placement and length of the posterior implant, four apical anatomic bone engagements are possible and can be classified as follows: (1) tuberosity, (2) tuberosity/pterygoid process, (3) tuberosity/pyramidal process, and (4) tuberosity/pyramidal process/pterygoid process. That is why is sometimes difficult to judge whether implants in a study were “pterygoid implants” or “tuberosity implants”. But basically, the difference in the placement of a “pterygoid implant” or a “tuberosity implant” lies mostly in the local anatomy present. Depending on the tuberosity’s dimension and quality, if it is adequate, an implant can be placed completely within the tuberosity; if not, implants can be angled and the apex can be made to engage the pyramidal process of the palate and/or the pterygoid process of the sphenoid bone [74], and these were the definitions adopted in the present review in order to distinguish the implant location. Therefore, it is possible to assume that it is possible to install implants in the maxillary tuberosity in a perpendicular position in relation to the occlusal plane (although not always), while the same is not possible with pterygoid implants.

Limitations of the present review include the fact that there was a considerable number of confounding factors. There was no information about how many implants were inserted and failed in several different conditions for most (if not all) of the studies. Studies reported the presence of patients with diabetes among the studied populations, as well as of smokers, patients with bruxism, and patients taking bisphosphonates. All these factors could have had a considerable impact on implant failure rates [75,76,77]. Moreover, there is a risk of bias in the comparison of survival between pterygoid and tuber implants due to imbalanced groups. Furthermore, the retrospective nature of many studies results in flaws manifested by the gaps in information. In addition, several studies presented small cohort sizes and short follow-ups.

## 5. Conclusions

Within the limitations of the present review, it is suggested that implants placed in the pterygoid process and in the maxillary tuberosity present a high 10-year CSR, although with lower survival rates for pterygoid in comparison to tuber implants. It is also suggested that pterygoid/tuber implants that are splinted with other implants may present higher survival rates than non-splinted implants.

## Figures and Tables

**Table 1:** Summarized data of the included studies.

Variable	Global	Pterygoid	Tuber
Implants/patients (n)	3446/2245	3053/1973	393/272
Men/women, n (%)	879 (43.4)/1148 (56.6)	740 (41.1)/1060 (58.9)	139 (61.2)/88 (38.8)
Not available	218	173	45
Age (years), mean ± SD (min–max)	58.0 ± 24.2 (14–90)	58.2 ± 25.1 (14–90)	57.0 ± 17.5 (38–82)
Implants per patient, n (%)			
1	1044 (46.5)	893 (45.3)	151 (55.5)
2	1201 (53.5)	1080 (54.7)	121 (44.5)
Follow-up (months), mean ± SD (min–max)	61.0 ± 36.3 (1–144)	62.9 ± 36.4 (1–123)	45.7 ± 31.2 (2–144)
Healing time, n (%)			
Immediate loading	1044 (30.3)	955 (31.3)	89 (22.6)
0.5–3 months	717 (20.8)	548 (17.9)	169 (43.0)
4–6 months	1643 (47.7)	1550 (50.8)	93 (23.7)
>6 months	42 (1.2)	0 (0)	42 (10.7)
Implant failure (n), failure/total (%)			
Implant level	220/3446 (6.4)	208/3053 (6.8)	12/393 (3.1)
Patient level	195/2245 (8.7)	183 /1973 (9.3)	12/272 (4.4)
Primary failure ^a^			
Immediate loading	-/36	-/25	-/1
0.5–3 months	14/26 (53.8)	10/20 (50.0)	4/6 (66.7)
4–6 months	83/158 (52.5)	83/153 (54.2)	0/5 (0)
>6 months	0	0	0
Time of failure (months), mean ± SD (min–max)	11.5 ± 14.2 (1–108)	11.8 ± 14.5 (1–108)	6.3 ± 6.0 (2–19)
Prosthetic rehabilitation ^c^, number of failed implants/number of implants used (%)			
Single crown	5/29 (17.2)	0 (0)	5/29 ^b^ (17.2)
Fixed partial prosthesis (2–6 units)	6/319 (1.9)	5/175 (2.9)	1/144 (0.7)
Fixed partial prosthesis (7–10 units)	2/30 (6.7)	2/21 (9.5)	0/9 (0)
Fixed full-arch prosthesis	172/2418 (7.1)	172/2289 (7.5)	0/129 (0)
Overdenture	4/36 (11.1)	4/36 (11.1)	0 (0)
Not available	614	532	82
Number of other maxillary implants, number of prostheses ^c^ (%)			
0	26 (5.2)	1 (0.3)	25 (21.0)
1	59 (11.9)	4 (1.1)	55 (46.2)
2	73 (14.7)	65 (17.2)	8 (6.7)
3	16 (3.2)	15 (4.0)	1 (0.8)
4	250 (50.4)	224 (59.4)	26 (21.8)
5	32 (6.5)	31 (8.2)	5 (0.8)
6	40 (8.1)	37 (9.8)	3 (2.5)

^a^ Failure up until prosthetic loading. ^b^ All 29 tuber implants used to support single crowns were loaded 4–6 months after implant installation, and all 5 failures happened only after prosthetic loaded had been applied, i.e., none of them were primary failures. ^c^ Information not available for all cases.

**Table 2:** Distribution of the prosthetic rehabilitation types by loading protocol, when the information was available for both variables.

Prosthetic Rehabilitation	Loading Protocol, n (%)		
	Immediate	Non-Immediate	Total
	Pterygoid		
Single crown	0 (0)	0 (0)	0 (0)
Fixed partial prosthesis (2–6 units)	0 (0)	175 (100)	175 (100)
Fixed partial prosthesis (7–10 units)	0 (0)	21 (100)	21 (100)
Fixed full-arch prosthesis	955 (41.7)	1334 (58.3)	2289 (100)
Overdenture	0 (0)	36 (100)	36 (100)
	Tuber		
Single crown	0 (0)	29 (100)	29 (100)
Fixed partial prosthesis (2–6 units)	5 (3.5)	139 (96.5)	144 (100)
Fixed partial prosthesis (7–10 units)	5 (55.6)	4 (44.4)	9 (100)
Fixed full-arch prosthesis	79 (61.2)	50 (38.8)	129 (100)
Overdenture	89 (28.6)	222 (71.4)	311 (100)

**Table 3:** Life table survival analysis showing the cumulative survival rate of pterygoid implants.

Interval Start Time (Years)	Number Entering Interval	Number Withdrawn during Interval	Number Exposed to Risk	Implant Failures	Survival Rate within Each Interval—ISR (%)	Cumulative Proportion Surviving at End of Interval—CSR (%)	SE
0	3053	66	3020.0	155	94.9	94.9	0.4
1	2832	278	2693.0	33	98.8	93.7	0.4
2	2521	169	2436.5	3	99.9	93.6	0.4
3	2349	255	2221.5	3	99.9	93.5	0.5
4	2091	279	1951.5	6	99.7	93.2	0.5
5	1806	872	1370.0	5	99.6	92.8	0.5
6	929	126	866.0	2	99.8	92.6	0.5
7	801	93	754.5	0	100.0	92.6	0.5
8	708	67	674.5	0	100.0	92.6	0.5
9	641	70	606.0	1	99.8	92.5	0.5
10	570	570	285.0	0	100.0	92.5	0.5

ISR—interval survival rate, CSR—cumulative survival rate, SE—standard error.

**Table 4:** Life table survival analysis showing the cumulative survival rate of implants in the maxillary tuberosity.

Interval Start Time (Years)	Number Entering Interval	Number Withdrawn during Interval	Number Exposed to Risk	Implant Failures	Survival Rate within Each Interval—ISR (%)	Cumulative Proportion Surviving at End of Interval—CSR (%)	SE
0	393	2	392.0	10	97.4	97.4	0.8
1	381	105	328.5	2	99.4	96.9	0.9
2	274	80	234.0	0	100.0	96.9	0.9
3	194	30	179.0	0	100.0	96.9	0.9
4	164	54	137.0	0	100.0	96.9	0.9
5	110	4	108.0	0	100.0	96.9	0.9
6	106	77	67.5	0	100.0	96.9	0.9
7	29	0	29.0	0	100.0	96.9	0.9
8	29	1	28.5	0	100.0	96.9	0.9
9	28	2	27.0	0	100.0	96.9	0.9
10	26	18	17.0	0	100.0	96.9	0.9
11	8	5	5.5	0	100.0	96.9	0.9
12	3	3	1.5	0	100.0	96.9	0.9

ISR—interval survival rate, CSR—cumulative survival rate, SE—standard error.

## Data Availability

All the data that resulted from this review are presented in the manuscript.

## Supplementary Materials

The following supporting information can be downloaded at: https://www.mdpi.com/article/10.3390/jcm13154544/s1, Table S1: Detailed data of the included studies; Kaplan–Meier curves: Figure S1: Cumulative survival of all implants; Figure S2: Comparison of survival between pterygoid and tuber implants; Figure S3: Comparison of survival between immediately and non-immediately loaded implants; Table S2: Life table survival analysis showing the cumulative survival rate of tuber/pterygoid implants when supporting single crowns; Table S3: Life table survival analysis showing the cumulative survival rate of tuber/pterygoid implants when supporting fixed partial prostheses; Table S4: Life table survival analysis showing the cumulative survival rate of tuber/pterygoid implants when supporting fixed full-arch prostheses; Table S5: Life table survival analysis showing the cumulative survival rate of tuber/pterygoid implants when supporting overdentures; Table S6: Quality assessment of the included studies, according to the National Institutes of Health (NIH); Table S7: Quality assessment of the included case reports, according to the CARE guidelines; PRISMA 2020 checklist.
